# Role of β3-Adrenergic Receptor in Bone Marrow Transplant as Therapeutical Support in Cancer

**DOI:** 10.3389/fonc.2022.889634

**Published:** 2022-06-08

**Authors:** Nicoletta Nastasi, Gennaro Bruno, Claudio Favre, Maura Calvani

**Affiliations:** ^1^Department of Health Sciences, University of Florence, Florence, Italy; ^2^Division of Pediatric Oncology/Hematology, Meyer Children’s Hospital, Florence, Italy

**Keywords:** β3-adrenoreceptor, cancer, hematopoietic stem cell (HSC), bone marrow, bone marrow transplant (BMT)

## Abstract

β3-adrenergic receptor (β3-AR) is the last β-adrenoceptor subtype identified. β3-AR is widely expressed and regulates numerous physiological processes, and it is also a potential target for the treatment of many diseases, including cancers. For some types of cancers, bone marrow transplant (BMT) represents a valid therapeutic support, especially in the case of the necessity of high-dose chemotherapy and radiotherapy. For a successful BMT, it is necessary that a donor’s hematopoietic stem cells (HSCs) correctly reach the staminal niche in the recipient’s bone marrow (BM) and contribute to restore normal hematopoiesis in order to rapidly repopulate BM and provide all the healthy blood cells of which the patient needs. Generally, it takes a long time. Control and accelerate homing and engraftment of HSCs could represent a helpful approach to avoid the complications and undesirable effects of BMT. The evidence that the β-adrenergic system has a role in the BM can be found in different studies, and this leads us to hypothesize that studying this field could be interesting to meliorate the most critical aspects of a BMT. Here, we collected the data present in literature about the role of β3-AR in the BM with the purpose of discovering a possible utility of β3-AR modulation in regulating HSC trafficking and hematopoiesis.

## β3-Adrenergic Receptor

Adrenergic receptors (ARs) are a class of membrane proteins that mediates the multiple metabolic and neuroendocrine effects of epinephrine and norepinephrine, the two neurotransmitters responsible for sympathetic nervous system (SNS)–induced “fight-or-flight” stress responses. Through the application of different functional and molecular techniques for studying ARs, it was possible to identify three major categories that differ for function and localization: α1-adrenoceptors, α2-adrenoceptors, and β-adrenoceptors (1). β-ARs (three subtypes identified, β1-, β2-, and β3-AR, with a fourth β4-AR remaining controversial) belong to the G-protein-coupled receptor (GPCR) family whose primary function is the transmission of information from the extracellular environment to the interior of the cell. The classical signal transduction *via* GPCRs depends on the receptor-mediated activation of heterodimeric G proteins. G proteins are classified into four families according to the α subunit, inhibitory G-protein (G_i_), stimulatory G protein (G_s_), G_12/13_, and G_q_ with different activities (2, 3). While β1-ARs are coupled to G_s_, β2-ARs are coupled to both G_s_ and G_i_, with predominant activation of the stimulatory one. β3-ARs are G_i_ protein coupled and additional intracellular signaling includes the activation of nitric oxide synthases (NOSs), activation of guanylate cyclase (GC), and formation of cGMP. Therefore, the type of the downstream effectors is determined by the subtype of β-AR that is activated (4, 5). In response to a continuous exposure to agonists, many GPCRs display desensitization, the fast loss of the ability to respond. Desensitization involves three distinct stages: receptor phosphorylation; interaction with scaffolding proteins; and internalization. Two protein families, the G protein-coupled receptor kinases (GRKs) and arrestins, were mainly found to mediate desensitization. The internalized receptors do not activate G proteins and may be recycled to the cell membrane or undergo degradation. Indeed, despite their high degree of sequence homology, each receptor displays characteristic properties: β2-ARs are the most susceptible to this process; β1-ARs undergo less rapid and efficient agonist-promoted internalization, whereas numerous studies suggest that β3-AR is resistant to these regulatory processes since β3-AR has no phosphorylation sites necessary for internalization (6–8). However, it has been recently reported in literature that β3-AR is susceptible to desensitization at multiple levels, including the downregulation of its mRNA, but this process occurs only in specific models and treatments conditions and, in any case, always in a less pronounced way compared to the other receptors (9). β3-AR is the last β-AR recognized. Initially, the β1- and β2-AR subtypes were identified, and only in the 1980s, a third β-AR was discovered primarily in rat adipose tissue (10, 11), and later, in 1989, Emorine et al. isolated the human gene encoding β3-AR (12). Ever since then, β3-AR expression has been reported in several human tissues, mainly in white and brown adipose tissue but also in the human gall bladder, colon, prostate, human heart, brain, and skeletal muscle (13, 14). It is now evident that β3-ARs are involved in the modulation of different physiological processes, such as lipolysis and thermogenesis, the regulation of bladder function, regulation of gastrointestinal motility, cardiac functions, and other numerous responses in health tissues. However, an aberrant expression of the β3-AR subtype has been recently shown in several cancers (15). These findings have led to the development of a large number of compounds modulating the activity of β-ARs for the clinical treatment of these numerous diseases. While the pharmacological characteristics of β1- and β2-ARs have been studied exhaustively, and a large number of clinically relevant agonists and antagonists have been characterized in competition binding and functional studies, less information is available about the pharmacological profile of the β3-AR. Regarding the endogenous agonists norepinephrine and epinephrine, according to the binding data, the β3-AR would be a noradrenergic receptor since it was observed to have a 30-fold higher affinity for norepinephrine over epinephrine (16). Instead, synthetic agonists can be distinguished in first-generation and second-generation compounds depending on the time of their discovery. BRL37344 is the first β3‐AR agonist developed and shows a very high potency in rodent β3‐AR relative to human β3‐AR, despite their shared structural homology of 80%–90%. CL316243 is a first-generation potent and highly selective β3-AR agonist. The second generation includes the aryloxypropanolamine CGP12177A, a 4-acylaminobenzene-sulfonamide derivative, the L-755507, Mirabegron (YM178), Amibegron (SR58611A), and other molecules that are currently being validated in phase II and III clinical trials, such as Solabegron (GW427353). Regarding antagonists, the drugs used most frequently are sotalol, alprenolol, carvedilol, metoprolol, atenolol, bisoprolol, SR59230A, and L-748,337, but their usefulness as selective antagonists of the β3-AR has not been fully clarified (17).

## β3-AR and Cancer

It was demonstrated that the SNS, through β-AR signaling, can influence the tumor biology, contributing to the regulation of several cellular processes that occur typically during the initiation and progression of cancer, for example, angiogenesis, cell trafficking, and inflammation (18–20). Among the β-AR subtypes, the specific role played by β3-AR in the oncological context has been analyzed in various laboratories through the evaluation of the β3-AR gene, mRNA, and protein expression and its blockade using selective β3-AR antagonists and through the siRNA silencing approach. Overall, accumulating lines of evidence point to a relevant function of β3-AR in the onset and progression of several types of cancer, such as vascular tumors (21), colon and breast cancers (22, 23), and especially melanoma (24). Recent studies have shown that β3-AR has a clear implication in the melanoma microenvironment because its expression was found upregulated not only in cancer cells but also in various accessory cells such as stromal, inflammatory, and vascular cells that sustain the tumorigenesis, and through them, the β3-AR causes pro-invasive, pro-angiogenic, and inflammatory effects. Moreover, β3-AR is able to increase the stemness potential and aggressiveness of melanoma cells favoring the malignancy progression, the effects reverted by the pharmacological use of selective β3-AR antagonists (25, 26). In a recent paper, the specific β3-AR antagonism has been useful in demonstrating the crucial role of β3-AR also in neuroblastoma: the treatment manages to decrease the proliferation rate of neuroblastoma cells and simultaneously increases neuronal differentiation. Moreover, it was seen that this switch from stemness to cell differentiation is regulated by β3-AR through a molecular interplay with sphingosine kinase 2 (SK2)/S1P receptor 2 (S1P2) axis, typically implicated in neuroblastoma biology (27). Interestingly, β3-AR expression has been reported also in human leukemia (28) where it appears to be involved in the survival of myeloid leukemia cells, especially under hypoxic conditions, to such an extent that β3-AR could be considered as a potential target also in this type of cancer, mostly to reduce chemoresistance, a phenomenon that occurs frequently in leukemic patients (29).

## Bone Marrow Transplant

The first human bone marrow transplant (BMT) was successfully experimented from 1950 to 1970 by a team under the leadership of Edward Donnall Thomas, whose work was later recognized with the Nobel Prize in Physiology and Medicine (30).

Starting from Thomas’s studies up to the present day, BMT has become the optimal therapy for a wide variety of hematological and nonhematological diseases, including leukemias (31), lymphomas, anemias, and immunological and genetic disorders (32).

Today, BMT is often helpful in oncological patients who need high-dose chemotherapy and/or radiation therapy. Chemotherapy and radiotherapy can target the tumors because cancer cells divide rapidly, more often than healthy cells. However, BM cells also divide frequently, so high-dose treatments can severely damage or destroy the patient’s BM. BMT represents a valid support to restore BM function that would otherwise be irreversibly altered; in this way, the patient can retrieve the blood cells they need to carry oxygen, fight infections, and prevent bleeding. The main objective of BMT is to substitute a defective system for a healthy one. Particularly, for the treatment of malignancies, it is important to eliminate malignant cells through the administration of cytotoxic drugs and/or radiation and the ability of donor cells to mediate the immunologic effect termed “graft-versus-tumor” against the malignant host cells; instead, for the treatment of immune deficiency or genetic diseases, it is necessary to replace defective host hematopoietic cells with normal hematopoietic stem cells (HSCs) capable of re-establishing hematopoiesis.

The main stages of a BMT are essentially two: the first is a recipient preparative treatment called “conditioning” regimens that usually lasts 1 week; the second consists of the transfusion of healthy BM.

The conditioning regimens are usually based on the administration of supralethal doses of total body irradiation and chemotherapeutic agents to the recipient with the goal of providing sufficient immunoablation to prevent graft rejection and reduce the tumor burden, increasing the chance of engraftment and decreasing the chance of relapse. However, as it was recognized that the graft-versus-tumor effects substantially contributed to the effectiveness of the transplant, reduced-intensity and nonmyeloablative conditioning regimens have been developed, making the transplant applicable to older and medically infirm patients (33).

In the second phase, the BM for transplantation is harvested by multiple aspirations from the donor’s iliac crests, processed and given to the recipient *via* intravenous infusion (34).

The period after a transplant is very crucial because it is characterized by 1 or 2 weeks of marked marrow aplasia in which the patient is at high risk of transmission of various pathogens or of the reactivation of latent infections (35); therefore, it is very important that a rapid BM repopulation occurs. BM repopulation is an active process that involves a complex interaction between many factors, molecules, and receptors in order to allow the so-called “homing” or rather the migration of the HSCs from the peripheral blood (PB) to the BM and their successful anchoring before proliferation (36). The HSCs are a rare population of multipotent stem cells with the dual capacity of self-renewal and differentiation to all hematopoietic lineages. The HSCs typically lodge in a specific compartment of the BM called a “staminal niche,” first mentioned by Schofield in 1978 to describe an inductive microenvironment that sustains and preserves the properties of HSCs (37). The niche provides all those factors that attract the HSCs within the BM, and it is unquestionable that the chemokine stromal-derived factor-1 (SDF-1) or Cxcl12 and its receptor CXCR4 axis plays a crucial role in the regulation of this mechanism. The administration of the granulocyte–colony stimulation factor (G-CSF) confirms the Cxcl12 role in HSCs maintaining within the BM because G-CSF is a glycoprotein able to stimulate the release of HSCs in the bloodstream through a complex mechanism that involves the induction of the proteolytic activity that cleaves Cxcl12 (38, 39).

It has been seen that the first population that regenerates the BM after a transplant is represented by fat cells, followed by immature, monotypic hematopoietic cells presumably originated from committed stem cells that gradually mature and enlarge. The normal marrow cellularity recovers gradually and, usually at least 3 weeks post-transplantation, must pass for the marrow to be approximately 50% normocellular and 8–12 weeks for a complete repopulation (40). In some people, it may take longer. It is now widely known that this trafficking of HSCs and other cells in and out of the BM occurs under the control of the SNS.

## β3-AR in Bone Marrow

The notion that BM is innervated by sympathetic fibers dates back many decades. Sympathetic nerve fibers enter in the BM with blood vessels that provide nutrient and oxygen, accompany the major arteries, and are distributed deeply into the substance of the marrow (41). It was hypothesized that these innervations provide a morphological basis for the neural modulation of the proliferation, differentiation, and migration of hematopoietic cells between the BM and the extramedullary sites. The confirmation of this hypothesis has come from the observation that any alteration of the SNS, for example, any stressful event that causes a progressive denervation or a premature aging of the innervations, simultaneously alters hematopoiesis and induces a dramatic remodeling of the hematopoietic niche (42). Some of the first evidence of sympathetic regulation of hematopoiesis came from studies on circadian rhythms, the daily oscillations that govern most biological processes synchronizing them with the natural light–darkness shift of day and night. Circadian activities are orchestrated by the suprachiasmatic nucleus, a tiny region of the brain in the hypothalamus that receives input signals from the retina through the retino-hypothalamic tract, processes the information, and generates adequate responses in different tissues, including BM, through the SNS innervations (43). The observation that continuous exposure to light or a “jet lag” (defined as a shift of 12 h) altered the number of HSCs in mouse BM, which indicates that photic cues could influence the trafficking of HSCs (44). *In vivo* experiments indicate that, in mice, a peak of HSCs has been detected in circulation during the daytime, whereas their BM homing occurs during the night, and the same trend has been also observed for ARs and their ligands (45). By examining the kinetics of daily light and dark cues at different *zeitgeber* time points of the day, Golan et al. identified two major functionally distinct peaks of HSC activity in the BM at 11:00 a.m. and 11:00 p.m., corresponding to norepinephrine and the tumor necrosis factor bursts that induce a metabolical reprogramming of HSCs. During the 11:00 a.m. peak, light induces an increase of the vascular permeability and resulting to accentuated HSC egress from the BM; in contrast, during the 11:00 p.m. peak, HSCs are primed to a state of retention in the BM. Consequently, the authors deduced that BM requires daily replenishment to ensure a homeostatic balance between mature blood cell production and stem cell maintenance (46). These HSC oscillations occur also in humans but in an antiphase with mice, being respectively diurnal and nocturnal species (47).

**Figure 1:**
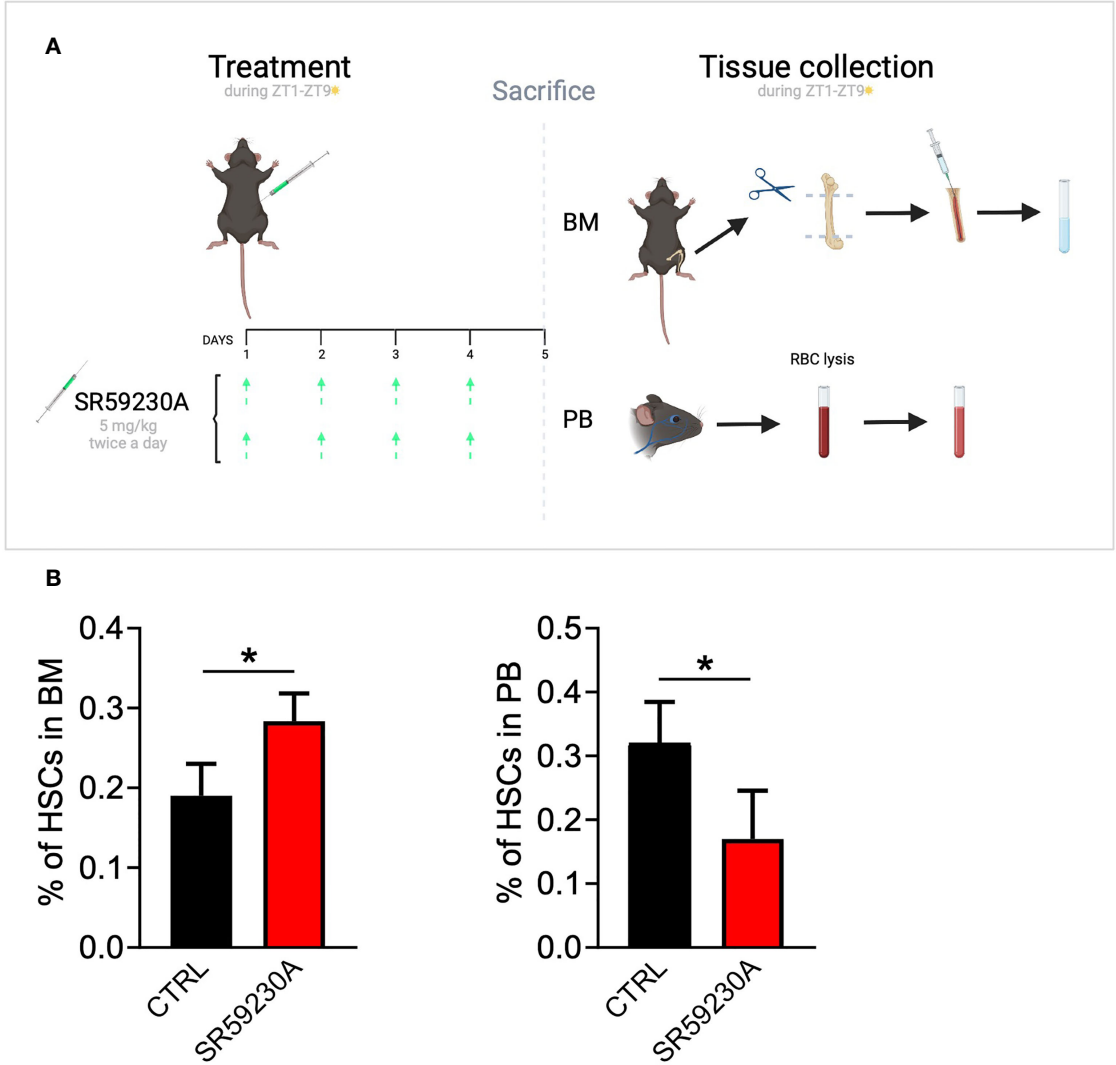
Study of β3-AR antagonism effect on HSC homing/egress. **(A)** *In vivo* model: C57BL/6 mice were treated with SR59230A 5 mg/kg twice a day for 4 days always during light hours (Zeitgeber time 1–9). Untreated mice were used as control. BM cells were obtained by flushing femur and tibia; PB cells were obtained by a retro-orbital sinus blood collection and the lysis of red blood cells. Samples were always collected during light hours (Zeitgeber time 1–9). **(B)** Flow cytometry analysis was performed by using a MACSQuant Analyzer 10 (Miltenyi Bio-tec). HSCs were identified as Lin-c-kit+Sca1+ cells. Results were reported as the mean ± SD of four replicates. *p <0,05 SR59230A vs. CTRL.

On our side, we experimentally tested the β3-AR inhibition effect on HSC homing/egress by using the selective antagonist SR59230A in an *in vivo* model (**Figure 1A**). Considering the daily oscillation of HSCs depending on light/darkness exposure, we administered the treatment and collected the samples always during the morning (Zeitgeber time 1–9). Through a cytofluorimetric analysis of HSCs in the BM and PB of the mice, we found that SR59230A administration increased the number of HSCs in the BM, instead of decreasing their number in the PB, confirming the aforementioned role of β3-AR in the HSCs trafficking in and out of the BM (**Figure 1B**).

Furthermore, in several studies, it was noted that the number of HSCs detectable in the bloodstream fluctuates in an antiphase with the expression of Cxcl12, the principal chemokine that, after the interaction with its receptor CXCR4 expressed by hematopoietic progenitor cells, dynamically regulates HSC attraction in the BM, and these rhythmic fluctuations are under the control of adrenergic signals delivered locally in the BM by nerves from the SNS.

To evaluate the exact contribution of ARs, it has been useful to use selective and non-selective adrenergic agonists and antagonists. Thanks to this strategy, it has been possible to ascertain that adrenergic signaling in the BM is mainly conveyed by β-ARs localized in many cellular components of the hematopoietic niche: β3-AR is restricted to marrow-adherent stromal cells producing Cxcl12, and β2-AR has been identified on osteoblast and hematopoietic progenitor cells. While the latter is involved in cell proliferation and bone remodeling, β3-AR is probably the principal contributor to the regulation of the SDF-1 expression level (48, 49). To gain more insight into the mechanisms that regulate the circadian fluctuations of Cxcl12, selective β3-AR agonists and antagonists were used and a decrease and increase in the Cxcl12 level was respectively found in a dose-dependent manner. It has been finally noticed that β3-AR acts also indirectly on the Cxcl12 through the regulation of the transcriptional factor Sp1. Sp1 typically binds to the Cxcl12 promoter at specific sites to induce its expression; indeed, the alterations of the Cxcl12 expression mirror the nuclear content of the transcription factor Sp1.

Since the Sp1 DNA-binding activity is enhanced by phosphorylation by the cAMP-dependent protein kinase (PKA), β3-AR can contribute to the degradation of Sp1-decreasing cAMP levels in the pathway that triggers after coupling to Gi proteins. All these data demonstrate how the Sp1 function is relevant for an efficient Cxcl12 expression and consequently how the β3-AR can affect the circulation of HSCs just acting on these elements (44, 51).

**Figure 2:**
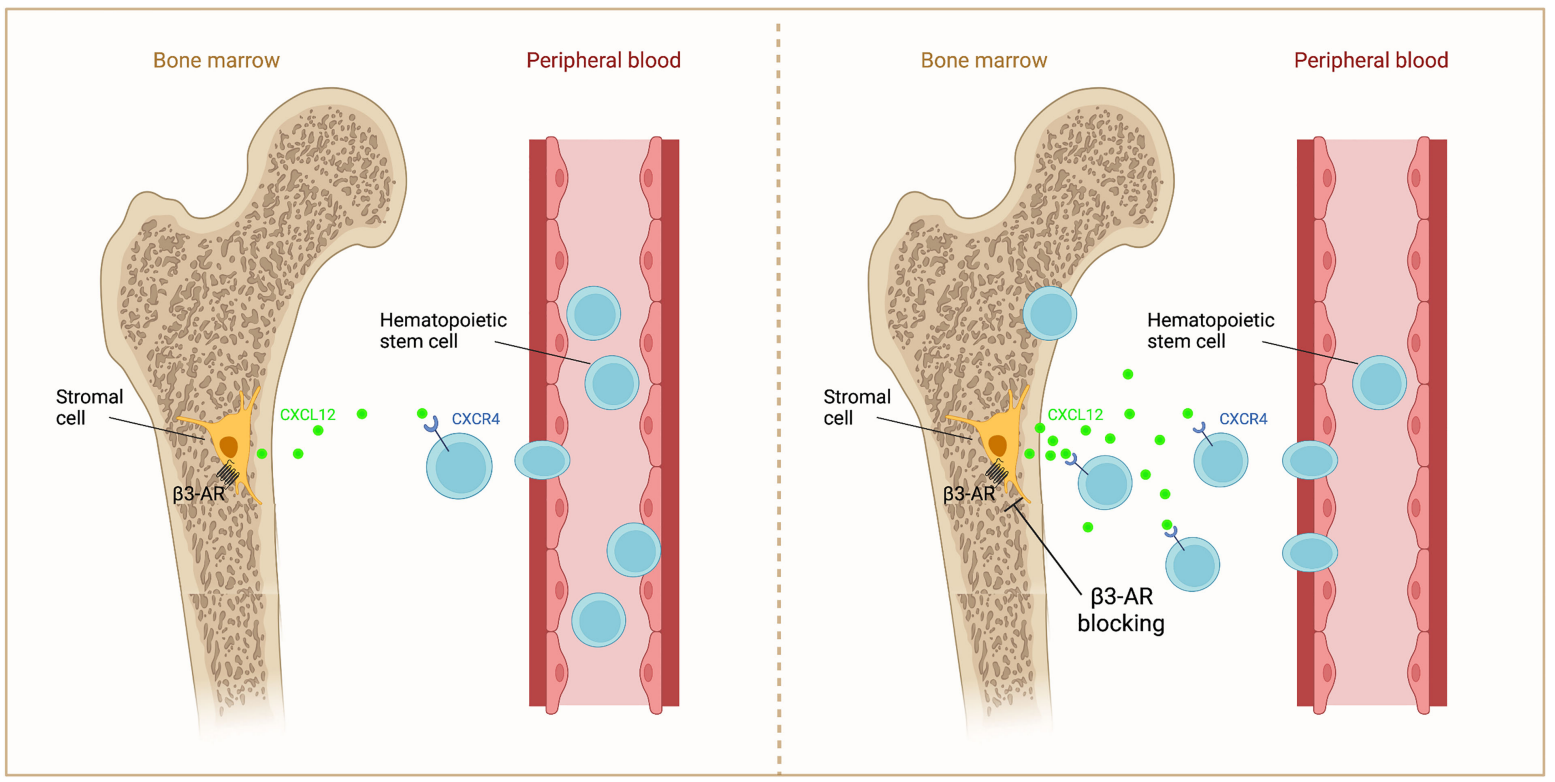
Schematic representation of β3-AR action on Cxcl12 in BM. β3-AR signaling decreases Cxcl12 secretion by stromal cells (left). β3-AR blocking increases Cxcl12 release, inducing the homing of HSCs to the BM (right).

Based on ample evidence, the inhibition of β3-AR could be used to support a crucial step for a successful BMT or the BM repopulation, through increasing the Cxcl12 release to stimulate the homing of HSCs (**Figure 2**).

## Hypothesis: Could β3-AR Modulation Regulate HSC Differentiation?

**Figure 3:**
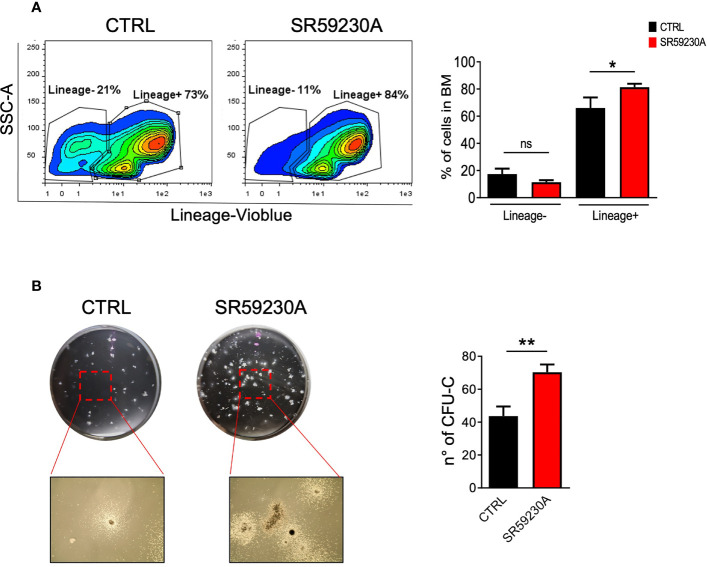
Study of the β3-AR antagonism effect on mouse BM cells (*in vivo* model as in **Figure 1A**). **(A)** Flow cytometry analysis of BM cells was performed by using MACSQuant Analyzer 10 (Miltenyi Bio-tec, Gladbach, Germany). Cells were stained with the Lineage cell detection cocktail and anti-biotin antibody. Results were reported as percentage (mean ± SD of three replicates) of Lineage-positive or -negative cells. **(B)** Images showing colonies obtained by performing a CFU assay with a MethoCult medium (Stemcell Technologies, Vancouver, Canada) and relative quantification in SR59230A and control conditions after 10 days of culture. Results were reported as the mean ± SD of three replicates. *p <0,05 **p <0,01 SR59230A vs. CTRL. ns, non significant.

After HSC homing, the engraftment step predicts stem cells to multiply and begin to make new, healthy blood cells. It usually takes several weeks before the number of blood cells returns to normal. During this period, patients must remain under close medical care and have a periodic follow-up appointment because of the risk of infections or other complications. Engraftment failure remains an important complication of BMT because of the high morbidity and mortality associated with this event. Two different clinical forms of defective engraftment have been distinguished: poor graft function (PGF) and graft failure (GF). PGF is characterized by the presence of an initial full donor engraftment. In the primary form, bone marrow cellularity remains low, and patients present persistent cytopenia. In the secondary form, a prompt recovery is followed by a progressive decrease in blood counts. Instead, GF occurs as the result of a classical alloreactive immune response mediated by residual host immunity persisting after the conditioning regimen (36, 50). Accelerating the engraftment and BM repopulation of committed cells may, therefore, represent a valid strategy for positive clinical outcomes in BMT. Considering this, we wondered if β3-AR could represent a useful target to be exploited in order to modulate these crucial phases of the BMT. With this purpose, we set up a colony-forming unit (CFU) assay and a cytofluorimetric analysis of BM cells obtained from mice treated and untreated with the β3-AR-selective antagonist SR59230A. Considering that primitive hematopoietic cells, including HSCs, do not express a variety of surface markers that are associated with the terminal maturation of specific blood cell types, for the cytofluorimetric analysis, we used a Lineage cell detection cocktail (a panel of monoclonal antibodies that recognize antigens on T cells, B cells, monocytes/macrophages, granulocytes, and erythrocytes) to distinguish immature cells (Lineage negative) from differentiated cells (Lineage positive). Comparing the percentages of Lineage-negative or -positive BM cells between control and SR59230A, we observed that the β3-AR blockade tends to decrease the percentages of lineage-negative cells, while it increases the number of lineage-positive cells (**Figure 3A**).

Moreover, we decided to perform a CFU assay on total mouse BM cells because it is the most widely used assay to get information about the frequency and types of progenitor cells present in the original cell population and their ability to proliferate and differentiate. As shown in **Figure 3B**, we counted a higher number of colonies produced by BM cells harvested from SR59230A-treated mice than in control. We can affirm that the administration of SR59230A augmented colony-forming capacity, indicative of increased progenitor cell proliferation and potential differentiation. This result is in line with that obtained from cytofluorimetric analysis because enhanced proliferation and differentiation of stem cells induced by β3-AR antagonism result in higher percentages of committed lineage-positive cells.

**Figure 4:**
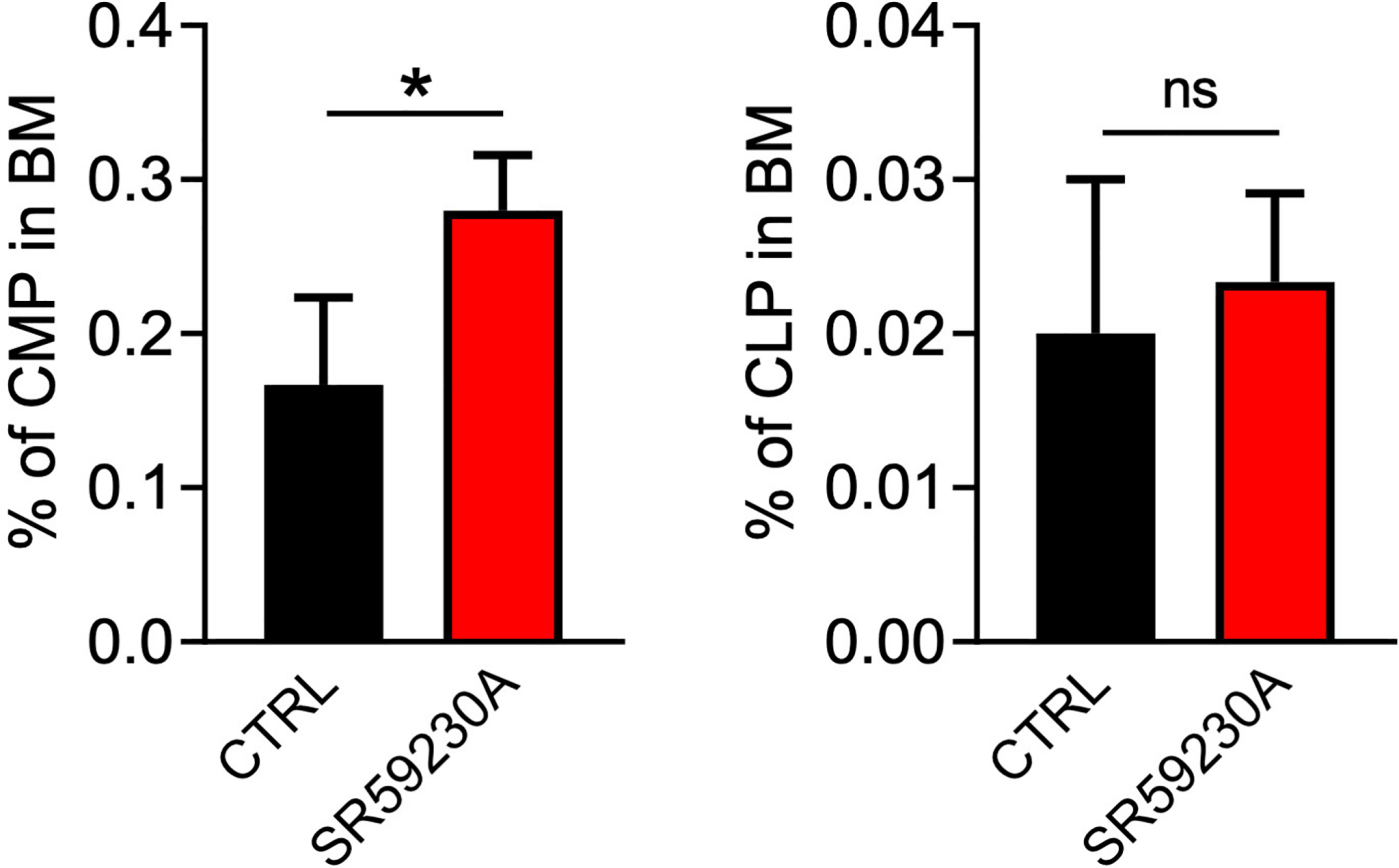
Study of the β3-AR antagonism effect on BM progenitor cells (*in vivo* model as in **Figure 1A**). Flow cytometry analysis was performed by using a MACSQuant Analyzer 10 (Miltenyi Bio-tec). CMP were identified as Lin^-^Sca1^-^CD117^+^CD16/32^-^CD34^+^, CLP as Lin^-^Sca1^low/int^CD117^low/int^CD127^+^. Results were reported as the mean ± SD of four replicates. *p <0,05 SR59230A vs. CTRL. ns, non significant.

To verify if our data truly proved an increased hematopoietic differentiation process in BM, rather than a redistribution of hematopoietic cells between BM, PB, and secondary lymphoid organs, we also analyzed the number of common myeloid progenitors (CMPs) and common lymphoid progenitors (CLPs) in the mouse BM, PB, and spleen. Cytofluorimetric analysis showed a higher number of CMPs in the BM of SR59230A-treated mice compared to control mice and no significative variation on the CLP number (**Figure 4**). However, we measured very few cytofluorimetric events and no significative differences in these progenitor numbers between the β3-AR antagonist treatment and control condition in PB and spleen (data not shown). These results confirm our hypothesis that the increased percentage of lineage-positive cells found in the BM of mice that received SR59230A administration was not due to a simple redistribution of cells between different districts but rather to an enhanced intramedullary differentiation.

## Conclusion

**Figure 5:**
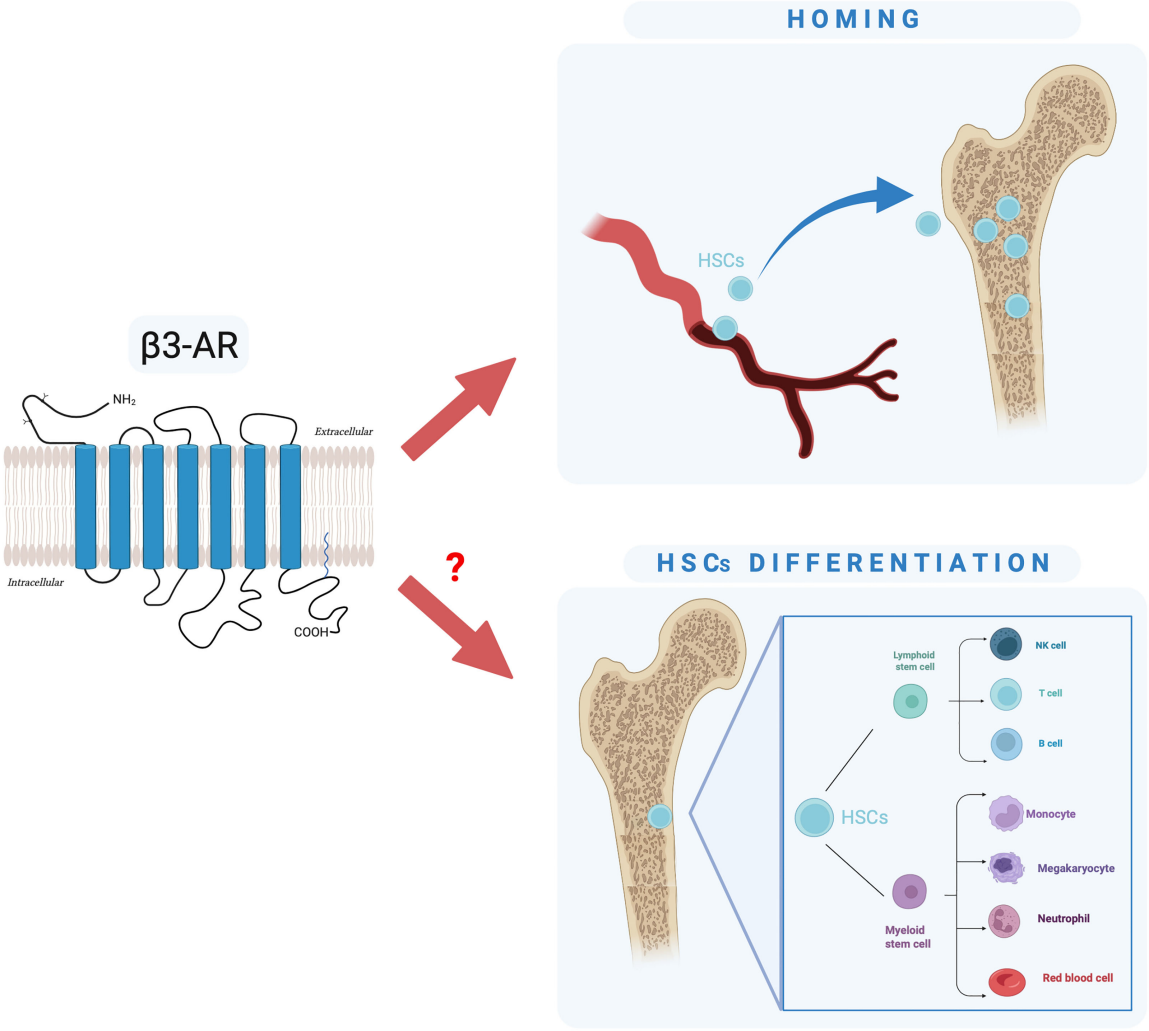
Schematic representation of our hypothesis: is β3-AR involved in HSC homing and differentiation?

Here, we have described a brief overview of the basic information regarding β3-AR and BMT, and we have speculated that targeting β3-AR could represent a new strategy to overcome complications related to BMT. Collectively, the studies reported here proved that the HSC release is not random or steady but rather follows a circadian rhythm regulated by “the molecular clock” of the SNS that exerts this function through the rhythmic secretion of catecholamines from the nerves in the BM, activation of the β3-AR, degradation of Sp1, and downregulation of Cxcl12. We focused on the possibility of a β3-AR inhibition to support the homing of HSCs in the BM, the step of a BMT in which the donor’s HSCs infused in the recipient patient must reach the staminal niche. In addition, with pilot experiments, we have put forward the hypothesis that β3-AR may also be involved in the HSC differentiation, an essential goal for a sustained long-term and effective engraftment phase of BMT (**Figure 5**). Our hypothesis leads the way to future explorations about the use of the β3-AR pharmacological modulation to ensure a successful BMT.

## Data Availability Statement

The raw data supporting the conclusions of this article will be made available by the authors, without undue reservation.

## Ethics Statement

The animal study was reviewed and approved by University of Florence and Italian Health Minister research permit no. 796/2021-PR.

## Author Contributions

NN wrote the review. NN and GB performed experiments. CF and MC supervised the project. All authors have read and agreed to the published version of the manuscript.

## Conflict of Interest

The authors declare that the research was conducted in the absence of any commercial or financial relationships that could be construed as a potential conflict of interest.

## Publisher’s Note

All claims expressed in this article are solely those of the authors and do not necessarily represent those of their affiliated organizations, or those of the publisher, the editors and the reviewers. Any product that may be evaluated in this article, or claim that may be made by its manufacturer, is not guaranteed or endorsed by the publisher.

## References

[1] Kirstein SL Insel PA. Autonomic Nervous System Pharmacogenomics: A Progress Report. Pharmacol Rev (2004) 56:31–52. doi: 10.1124/pr.56.1.2 15001662

[2] Hodavance SY Gareri C Torok RD Rockman HA. G Protein-Coupled Receptor Biased Agonism. J Cardiovasc Pharmacol (2016) 67(3):193–202. doi: 10.1097/FJC.0000000000000356 26751266 PMC4783281

[3] Neves SR Ram PT Iyengar R. G Protein Pathways. Science (2002) 296(5573):1636–9. doi: 10.1126/science.1071550 12040175

[4] Randy A. Hall. β-Adrenergic Receptors and Their Interacting Proteins. Semin Cell Dev Biol (2004) 15(3):281–8. doi: 10.1016/j.semcdb.2003.12.017 15125891

[5] Evans BA Sato M Sarwar M Hutchinson DS Summers RJ. Ligand-Directed Signalling at β-Adrenoceptors. Br J Pharmacol (2010) 159(5):1022–38. doi: 10.1111/j.1476-5381.2009.00602.x PMC2839261 20132209

[6] Nantel F Bonin H Emorine LJ Zilberfarb V Strosberg AD Bouvier M. The Human β3-Adrenergic Receptor is Resistant to Short Term Agonist- Promoted Desensitization. Mol Pharmacol (1993) 43:548–55. 8386307

[7] Curran PK Fishman PH. Endogenous β3- But Not β1-Adrener- Gic Receptors are Resistant to Agonist-Mediated Regulation in Human SK- N-MC Neurotumor Cells. Cell Signalling (1996) 8:355–64. doi: 10.1016/0898-6568(96)00068-X 8911684

[8] Milano S Gerbino A Schena G Carmosino M Svelto M Procino G. Human β3-Adrenoreceptor is Resistant to Agonist-Induced Desensitization in Renal Epithelial Cells. Cell Physiol Biochem (2018) 48:847–62. doi: 10.1159/000491916 30032151

[9] Okeke K Angers S Bouvier M Michel MC. Agonist-Induced Desensitisation of β3-Adrenoceptors: Where, When, and How? Br J Pharmacol (2019) 176(14):2539–58. doi: 10.1111/bph.14633 PMC6592865 30809805

[10] Tan S Curtis-Prior PB. Characterization of the Beta- Adrenoceptor of the Adipose Cell of the Rat. Int J Obes (1983) 7(5):409–14. 6139348

[11] Arch JR Ainsworth AT Cawthorne MA Piercy V Sennitt MV Thody VE. Atypical be-Ta-Adrenoceptor on Brown Adipocytes as Target for Anti- Obesity Drugs. Nature (1984) 309(5964):163–5. doi: 10.1038/309163a0 6325935

[12] Emorine LJ Marullo S Briend-Sutren MM Patey G Tate K Delavier-Klutchko C. Molecular Characterization of the Human Beta 3-Adrenergic Receptor. Science (1989) 245(4922):1118–21. doi: 10.1126/science.2570461 2570461

[13] Ursino MG Vasina V Raschi E Crema F De Ponti F. The β3-Adrenoceptor as a Therapeutic Target: Current Perspectives. Pharmacol Res (2009) 59(4):221–34. doi: 10.1016/j.phrs.2009.01.002 19429463

[14] Coman OA Panescu H Ghita I Badararu A Fulga I. Beta 3 Adrenergic Receptors: Molecular, Histological, Functional and Pharmacological Approaches. Romanian J Morphol Embryol (2009) 50(2):169–79. 19434307

[15] Schena G Caplan MJ. Everything You Always Wanted to Know About β3-AR (But Were Afraid to Ask). Cells (2019) 8(4):357. doi: 10.3390/cells8040357 PMC6523418 30995798

[16] Hoffmann C Leitz MR Oberdorf-Maass S Lohse M Klotz KN. “Comparative Pharmacology of Human β-Adrenergic Receptor Subtypes—Characterization of Stably Transfected Receptors in CHO Cells”. Naunyn-Schmiedeberg’s Arch Pharmacol (2004) 369:151–9. doi: 10.1007/s00210-003-0860-y 14730417

[17] Perrone MG Bleve L Santandrea E Vitale P Niso M Scilimati A. The Tertiary Amine Nitrogen Atom of Piperazine Sulfonamides as a Novel Determinant of Potent and Selective Beta3-Adrenoceptor Agonists. ChemMedChem (2009) 4(12):2080–97. doi: 10.1002/cmdc.200900292 19882697

[18] Tilan J Kitlinska J. Sympathetic Neurotransmitters and Tumor Angiogenesis—Link Between Stress and Cancer Progression. J Oncol (2010) 2010:539706. doi: 10.1155/2010/539706 20508839 PMC2874925

[19] Tang J Li Z Lu L Cho CH. β-Adrenergic System, a Backstage Manipulator Regulating Tumour Progression and Drug Target in Cancer Therapy. Semin Cancer Biol (2013) 23:533–42. doi: 10.1016/j.semcancer.2013.08.009 24012659

[20] Cole SW Sood AK. Molecular Pathways: Beta-Adrenergic Signaling in Cancer. Clin Cancer Res (2012) 18(5):1201–6. doi: 10.1158/1078-0432.CCR-11-0641 PMC3294063 22186256

[21] Chisholm KM Chang KW Truong MT Kwok S West RB Heerema-McKenney AE. β-Adrenergic Receptor Expression in Vascular Tumors. Mod Pathol (2012) 25(11):1446–51. doi: 10.1038/modpathol.2012.108 22743651

[22] Perrone MG Notarnicola M Caruso MG Tutino V Scilimati A. Upregulation of Beta3- Adrenergic Receptor mRNA in Human Colon Cancer: A Preliminary Study. Oncology (2008) 75:224–9. doi: 10.1159/000163851 18852493

[23] Amaya MA Belmont CN Diab A Trevino N Villanueva R Rains G. Use of non-Selective Beta-Blockers is Associated With Decreased Tumor Proliferative Indices in Early-Stage Breast Cancer. Oncotarget (2017) 8:6446–60. doi: 10.18632/oncotarget.14119 PMC5351644 28031536

[24] Dal Monte M Casini G Filippi L Nicchia GP Svelto M Bagnoli P. Functional Involvement of B3- Adrenergic Receptors in Melanoma Growth and Vascularization. J Mol Med (2013) 91:1407–19. doi: 10.1007/s00109-013-1073-6 23907236

[25] Calvani M Floriane P Comito G Taddei ML Moretti S Innocenti S. Norepinephrine Promotes Tumor Microenvironment Reactivity Through β3-Adrenoceptors During Melanoma Progression. Oncotarget (2015) 6(7):4615–32. doi: 10.18632/oncotarget.2652 PMC4467103 25474135

[26] Calvani M Bruno G Dabraio A Subbiani A Bianchini F Fontani F. β3-Adrenoreceptor Blockade Induces Stem Cells Differentiation in Melanoma Microenvironment. Int J Mol Sci (2020) 21(4):1420. doi: 10.3390/ijms21041420 PMC7073111 32093135

[27] Bruno G Cencetti F Pini A Tondo A Cuzzubbo D Fontani F. β3-Adrenoreceptor Blockade Reduces Tumor Growth and Increases Neuronal Differentiation in Neuroblastoma *via* SK2/S1P 2 Modulation. Oncogene (2020) 39(2):368–84. doi: 10.1038/s41388-019-0993-1 PMC6949192 31477835

[28] Lamkin DM Sloan EK Patel AJ Chiang BS Pimentel MA Ma JC. Chronic Stress Enhances Progression of Acute Lymphoblastic Leukemia *via* β-Adrenergic Signaling. Brain Behav Immun (2012) 26:635–41. doi: 10.1016/j.bbi.2012.01.013 PMC3322262 22306453

[29] Calvani M Dabraio A Bruno G De Gregorio V Coronnello M Bogani C. β3-Adrenoreceptor Blockade Reduces Hypoxic Myeloid Leukemic Cells Survival and Chemoresistance. Int J Mol Sci (2020) 21(12):4210. doi: 10.3390/ijms21124210 PMC7352890 32545695

[30] Thomas ED Lochte HL Lu WC Ferrebee JW. Intravenous Infusion of Bone Marrow in Patients Receiving Radiation and Chemotherapy. New Engl J Med (1957) 157(11):491–6. doi: 10.1056/NEJM195709122571102 13464965

[31] Fagioli F Zecca M Locatelli F Lanino E Uderzo C Di Bartolomeo P. Allogeneic Stem Cell Transplantation for Children With Acute Myeloid Leukemia in Second Complete Remission. J Pediatr Hematol Oncol (2008) 30(8):575–83. doi: 10.1097/MPH.0b013e31816e2342 18799933

[32] Khaddour K Hana CK Mewawalla P. Hematopoietic Stem Cell Transplantation (Bone Marrow Tranplant). Treasure Island FL: StatPearls Publishing) (2020). 30725636

[33] Gyurkocza B Sandmaier BM. Conditioning Regimens for Hematopoietic Cell Transplantation: One Size Does Not Fit All. Blood (2014) 124(3):344–53. doi: 10.1182/blood-2014-02-514778 PMC4102707 24914142

[34] Babic A Trigoso E. Chapter 5: Cell Source and Apheresis. In: Kenyon M Babic A, editors. The European Blood and Marrow Trans-Plantation Textbook for Nurses: Under the Auspices of EBMT. Cham (CH: Springer (2018). 31314221

[35] Angheben A Giaconi E Menconi M Casazza G Najajreh M Anselmi M. Reactivation of Chagas Disease After a Bone Marrow Transplant in Italy: First Case Report. Blood Transfus (2012) 10(4):542–4. doi: 10.2450/2012.0015-12 PMC3496236 22790268

[36] Caocci G Greco M La Nasa G. Bone Marrow Homing and Engraftment Defects of Human Hem-Atopoietic Stem and Progenitor Cells. Mediterr J Hematol Infect Dis (2017) 9(1):e2017032. doi: 10.4084/MJHID.2017.032 28512561 PMC5419183

[37] Schofield R. The Relationship Between the Spleen Colony-Forming Cell and the Haemopoietic Stem Cell. Blood Cells (1978) 4:7–25. 747780

[38] Levesque JP Winkler IG. Mobilization of Hematopoietic Stem Cells: State of the Art. Curr Opin Organ Transplant (2008) 13(1):53–8. doi: 10.1097/MOT.0b013e3282f42473 18660707

[39] Asri A Sabour J Atashi A Soleimani M. Homing in Hematopoietic Stem Cells: Focus on Regulatory Role of CXCR7 on SDF1a/CXCR4 Axis. EXCLI J (2016) 15:134–43. doi: 10.17179/excli2014-585 PMC4827072 27092040

[40] Riley RS Idowu M Chesney A Zhao S McCarty J Lamb LS. Hematologic Aspects of Myeloablative Therapy and Bone Marrow Transplantation. J Clin Lab Anal (2005) 19:47–79. doi: 10.1002/jcla.20055 15756708 PMC6807857

[41] Felten DL Felten SY Carlson SL Olschowka JA Livnat S. Noradrenergic and Peptidergic Innervation of Lymphoid Tissue. J Immunol (1985) 135(2 Suppl):755s–65s. 2861231

[42] Maryanovich M Zahalka AH Pierce H Pinho S Nakahara F Asada N. Adrenergic Nerve Degeneration in Bone Marrow Drives Aging of the Hematopoietic Stem Cell Niche. Nat Med (2018) 24(6):782–91. doi: 10.1038/s41591-018-0030-x PMC6095812 29736022

[43] Del Toro R Méndez-Ferrer S. Autonomic Regulation of Hematopoiesis and Cancer. Haematologica (2013) 98(11):1663–6. doi: 10.3324/haematol.2013.084764 PMC3815164 24186311

[44] Mendez-Ferrer S Lucas D Battista M Frenette PS. Haematopoietic Stem Cell Release is Regulated by Circadian Oscillations. Nature (2008) 452:442–7. doi: 10.1038/nature06685 18256599

[45] García-García A Korn C García-Fernández M Domingues O Villadiego J Martín-Pérez D. Dual Cholinergic Signals Regulate Daily Migration of Hematopoietic Stem Cells and Leukocytes. Blood (2019) 133(3):224–36. doi: 10.1182/blood-2018-08-867648 PMC6449569 30361261

[46] Golan K Kumari A Kollet O Khatibi-Massalha E Subramaniam MD Ferreira ZS. Daily Onset of Light and Darkness Differentially Controls Hematopoietic Stem Cell Differentiation and Maintenance. Cell Stem Cell (2018) 23(4):572–85.e7. doi: 10.1016/j.stem.2018.08.002 30174297

[47] García-García A Méndez-Ferrer S. The Autonomic Nervous System Pulls the Strings to Coordinate Circadian HSC Functions. Front Immunol (2020) 11:956. doi: 10.3389/fimmu.2020.00956 32508835 PMC7251159

[48] Maestroni GJM. Adrenergic Modulation of Hematopoiesis. J Neuroimmune Pharmacol (2020) 15:82–92. doi: 10.1007/s11481-019-09840-7 30762159

[49] Méndez-Ferrer S Battista M Frenette PS. Cooperation of Beta2- and Beta3-Adrenergic Receptors in Hematopoietic Progenitor Cell Mobilization. Ann N Y Acad Sci (2010) 1192:139–44. doi: 10.1111/j.1749-6632.2010.05390.x PMC4106131 20392229

[50] Giudice A Caraglia M Marra M Montella M Maurea N Abbruzzese A. Circadian Rhythms, Adrenergic Hormones and Trafficking of Hematopoietic Stem Cells. Expert Opin Ther Targets (2010) 14(5):567–75. doi: 10.1517/14728221003769887 20350049

[51] Masouridi-Levrat S Simonetta F Chalandon Y. Immunological Basis of Bone Marrow Failure After Allogeneic Hematopoietic Stem Cell Transplantation. Front Immunol (2016) 7:362. doi: 10.3389/fimmu.2016.00362 27695456 PMC5025429

